# A Controlled Atmosphere Chamber

**DOI:** 10.6028/jres.067A.028

**Published:** 1963-06-01

**Authors:** Charles L. Gordon, Rolf B. Johannesen

## Abstract

An inert atmosphere chamber for the transfer of reactive materials is described. It has the advantages of being inexpensive, easily cleaned, and can be evacuated.

## 1. Introduction

Many chemical compounds must be kept under an inert atmosphere while being transferred or sampled. Descriptions of a number of different inert- atmosphere transfer chambers or so-called dry-boxes have been published. These can be classed in three types. The simplest is the inexpensive transfer chamber maintaining protection from the ordinary atmosphere by a continuous flow of an appropriate inert gas. Suitably equipped plastic bags [1, 2, 3, 4, 5],^1^ a section of pipe [6], the open bell jar [7], a reaction vessel [8] and a refrigerator shell [9] are of this type.

A second type is the dry-box with a constantly maintained atmosphere [10, 11, 12, 13, 14] which may be regenerated and recirculated. These dry boxes are most elaborate and include electrical outlets and even gas cocks. They are not easily purged when some organic vapors are introduced. A device sometimes used to eliminate the long flushing time required is to expel the contaminated vapor in the chamber by an inflating balloon [11].

A third type can be evacuated so that repurification of the inert gas or long equilibration periods [15, 16] can be avoided.

The chamber described here is less expensive to build than many of those described in the literature cited or commercially available and has a greater versatility for small operations in the laboratory. It is of the evacuable type mentioned above. For the occasional transfer and bottling of various samples this type of chamber offers the least expensive method in which the atmosphere can be purged completely when vapors from one sample would contaminate the next material to be transferred.

Another advantage of the chamber described here is the ease with which the whole chamber or any portions of it can be disassembled and cleaned. The bell jar itself can be further protected against surface deposits by lining it with a removable plastic film [17].

## 2. Chamber

**Figure 1:**
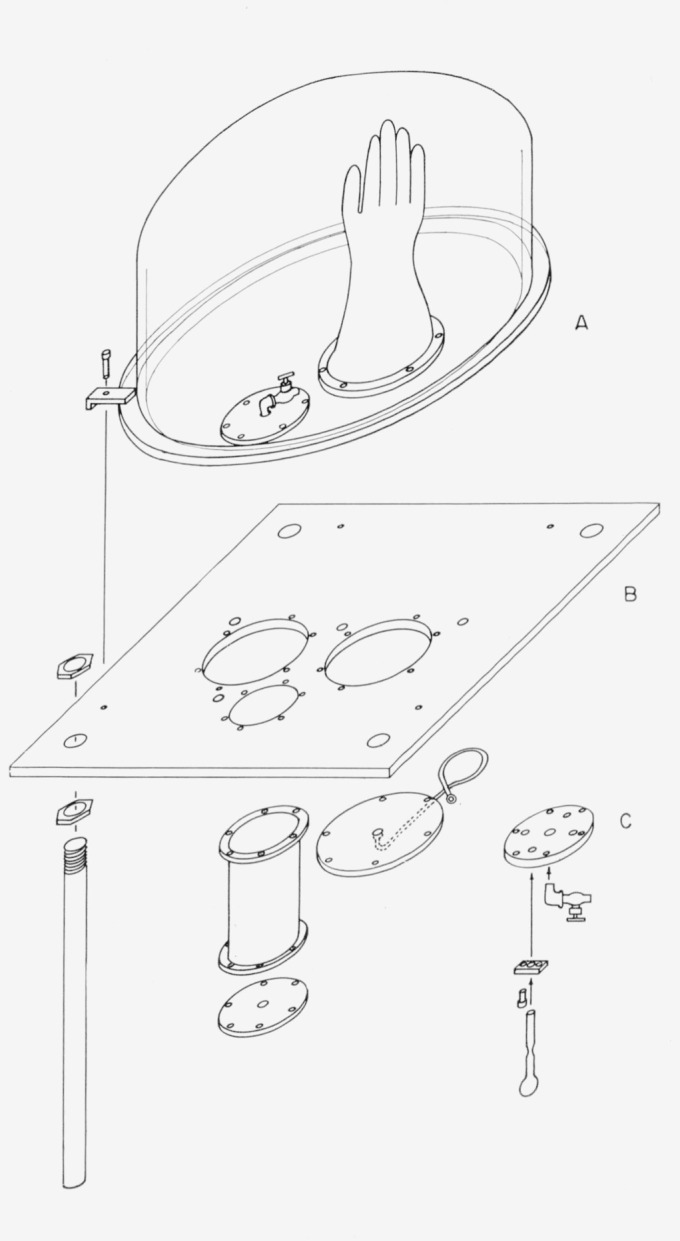
Assembly of the inert atmosphere chamber. Parts which are duplicated are here shown only once.
Plastic bell jar.Base plate.Sampling attachment.

The chamber is illustrated in figure 1. It consists of two main parts, the commercially available plastic bell jar, A, which measures 16 in. inside diameter and 14 in. inside height, and the base plate, B, which is a 20 in. square brass plate ½ in. thick.

The base plate has several openings: two 5-in. diameter holes for the gloves, one 3-in. hole for an antechamber, and four holes threaded for ¼**-in. pipe, each closed by a needle valve for control of evacuation and admission of the inert atmosphere. A design using the base plate in a vertical position with a shelf on it for a working area is more convenient. However, the sampling technique described later can not be used.

The base plate has four legs (18 in. long) attached at its corners, which hold it up sufficiently high above the table top that the operator’s arms can be freely moved about under the chamber while his hands are inside the chamber. Also sufficient room is available for the introduction from below of equipment into the antechamber. All the flat metal-to-metal seals are gasketed by cutouts from a 1/16 in. neoprene sheet.

On both the top and bottom faces of the base plate, sets of six equally spaced, blind-tapped (¼–20) holes are provided, surrounding each of the glove openings and the ante-chamber opening. These tapped holes are for the ¼–20×½ Hex Socket Head Cap Screws used to fasten the glove retaining rings and the inside antechamber plate on the top of the base plate. The tapped holes on the underside are for fastening the glove port cover plates and the antechamber by the same means.

Neoprene gloves [18, 19] are clamped over the glove holes by means of ¼ in. thick brass rings of 6 in. outside, and 5 in. inside, diameter.

## 3. Atmosphere

For many uses oil-pumped helium, or argon, direct from the tanks is sufficiently inert. In our use for transferring samples of titanium salts, the gas needed to be purified further. Therefore, a tower filled with a mixture of glass beads and titanium trichloride in the lower half and a mixture of glass beads and potassium hydroxide pellets in the upper half was inserted in the gas line, the gas passing from bottom to top. This was followed by a trap packed loosely with glass wool which was immersed in liquid nitrogen (for final drying of helium or hydrogen, only).

For short periods the inert gas in the chamber can be maintained by a filled balloon [20] attached to the exit valve.

## 4. Operation of the Chamber

The gloves are folded on the top of the base plate and the antechamber plate is placed over the antechamber opening. Any apparatus which is to be needed and which can withstand evacuating is placed on the base plate and the bell jar is placed in position. The lids for the glove openings are secured in position under the base plate and are attached to the vacuum line. The antechamber with its outside lid is made fast to the bottom of the base plate. The whole apparatus and antechamber is evacuated, flushed once with dry helium, reevacuated and a flow of dry helium is then put through the chamber and allowed to escape through the antechamber to the air.

Small pieces of apparatus which cannot stand evacuation are introduced to the chamber through the antechamber, allowing the apparatus to remain in the antechamber sufficiently long to flush out all air. When using the antechamber in this way there is a distinct advantage in using a gas lighter than air so that the density difference is favorable for keeping air from entering the antechamber.

In some instances when hydrogen or helium is used and where the sweeping out of the residual air is not critical the gas can be admitted to the top of the bell jar by placing a bent tube on the gas inlet opening. Slow displacement of the air from the top of the chamber downward is thus accomplished.

## 5. Sampling Attachment and Technique

A disk 4⅛ in. in diameter was made from ½ in. thick brass plate with six ¼ in. holes matching the set used to attach the antechamber. Four 10-mm holes and four small plates for clamping were made as indicated in the figure at C. The clamping gasket was made by carefully cutting small rectangles of neoprene sheet to match these rectangular clamping plates and cutting a hole in each slightly under 10-mm in diameter by means of a cork borer.

A stock of 10-mm tubing which would just pass through the 10-mm holes was obtained by selection from ordinary stock tubing. Pieces of this tubing about 8 in. in length were cleaned and a bulb with a breakable tip blown on one end. When positioned in the clamp, clamping gasket, and attachment plate, the seal of the tube to plate was made vacuum tight by tightening the clamp.

With the four hole plate as shown, four samples could be sealed off at a time. This can be done as follows: Four tubes marked at both ends are weighed and clamped in the plate with the open ends uppermost and projecting not more than ⅜ in. above the plate. The plate with its gasket is next attached to the base plate in place of the antechamber and the valve connected to the vacuum line. The inside antechamber plate with its gasket (and with its valve closed) is placed over the antechamber hole which now has the sample tubes projecting into it from below. The valve in the sample tube plate is opened and the sample tubes evacuated, after which the inert atmosphere is introduced by closing the valve to the vacuum and opening the inside antechamber plate valve. The inside plate is released and lifted away from the antechamber opening. The samples are then introduced into the tubes after which the pressure in the chamber is reduced and the sample tubes sealed off. The sample weight is obtained as the difference between the sum of the weights of the two parts of the sealed off tube and the original weight of the tube, all corrected for buoyancy.

A controlled atmosphere chamber constructed as described above has been used for about 6 years in these laboratories and has proved to be quite satisfactory in the handling of materials which are susceptible to contamination by moisture or oxygen. Even such sensitive material as finely divided titanium trichloride has been handled without contamination. The ease of cleaning spilled materials and the rapidity with which the apparatus can be reassembled, as mentioned above, have enhanced the value in our day to day use.

## Footnotes

1 Figures in brackets indicate the literature references at the end of this paper.
